# Dosimetric superiority of IMRT with jaw tracking technique for whole esophagus and T-shaped field radiotherapy in advanced esophageal cancer

**DOI:** 10.1371/journal.pone.0202628

**Published:** 2018-09-05

**Authors:** Liwan Shi, Youqun Lai, Shanyu Chen, Lirong Fu, Qin Lin

**Affiliations:** Department of Radiation Oncology, Xiamen Cancer Hospital, The First Affiliated Hospital of Xiamen University, Xiamen, PR China; North Shore Long Island Jewish Health System, UNITED STATES

## Abstract

**Purpose:**

For whole esophagus and T-shaped field radiotherapy using intensity modulated radiotherapy (IMRT) technique in advanced esophageal cancer, lower absorbed doses to lung and heart remains a challenge. The aim of this study was to investigate the dosimetric superiority in IMRT plans with jaw tracking technique for whole esophagus radiotherapy.

**Methods and materials:**

Thirty-two patients with esophageal cancer were subjected to IMRT treatment plans using Eclipse treatment planning system. For every patient, four different plans were generated with six gantry angles: six large fields IMRT plans with fixed jaw (6F-IMRT), six large fields IMRT plans with jaw tracking technique (6F-IMRT-T), twelve small fields IMRT plans with fixed jaw (12F-IMRT), and twelve small fields IMRT plans with jaw tracking technique (12F-IMRT-T). Dosimetric evaluation was assessed for all plans.

**Results:**

For every technique, there were no differences in planning target volume (PTV) coverage and conformity. 6F-IMRT-T plans could significantly reduce lung irradiation with 7.9% (*P*<0.001) reduction in V5_lung_ and 2.5% (*P*<0.001) reduction in V20 _lung_ respectively compared to 6F-IMRT plans. 12F-IMRT-T plans resulted in superior plans compared to 12-IMRT plans with a reduction of 2.9% (*P*<0.001) in V5_lung_ and 0.9% (*P*<0.001) in V20 _lung_, respectively. For heart irradiation, 6F-IMRT-T and 12F-IMRT-T plans were slightly superior to 6F-IMRT and 12-IMRT plans respectively with a reduction of 1.1 Gy and 0.5 Gy in the respective mean doses.

**Conclusions:**

By the use of jaw tracking technique, the IMRT plans resulted in further lung and heart sparing compared to fixed jaw plans for radiotherapy in esophageal cancer.

## Introduction

Esophageal cancer is cancer arising from the esophagus and is the eighth-most common cancer in the world [1]. Currently concurrent chemoradiotherapy is commonly used especially in the treatment of advanced, unresectable esophageal cancer [2–4]. Radiation therapy is one of the main modalities for the treatment of esophageal cancer; however the risk of radiation-induced toxicity (expecially radiation pneumonitis) may be increased due to the large target volume irradiation and combined- chemotherapy.

Radiation pneumonitis is the most common complication of esophageal radiation, and the incidence is 10–20% in the clinic [5]. Various dosimetric parameters of lung (e.g., V5, V20 and mean dose) usually have a strong correlation with radiation pneumonitis risk [6]. To reduce the radiation dose value of surrounding normal tissues and minimize the risk of radiation-induced toxicity, several investigators have studied various and sophisticated techniques. Louis Fenkell *et al*. [7] have compared plan quality (e.g., target coverage, normal tissues sparing) of IMRT and 3D conformal radiotherapy (3D-CRT) for esophageal cancer irradiation, and showed that IMRT plans resulted in superior normal tissues sparing. In addition, there are some other techniques for treatment of esophageal cancer, such as hybrid IMRT [8], volumetric modulated arc therapy (VMAT) [9–10] and VMAT with flattening filter-free beams [11]. In recent years, the linear accelerator of TrueBeam system (Varian Medical Systems, Paolo Alto, CA) with jaw tracking function has been widely used in clinical tumor treatment. Sarah Joy *et al*. [12] reported the dosimetric effects of jaw tracking in step-and-shoot IMRT, and concluded that the step-and-shoot IMRT with jaw tracking technique was probably not clinically significant. The jaw tracking technique in IMRT or VMAT plans have been compared with static jaw technique by some investigators, and showed that jaw tracking technique can provide superior normal tissues sparing [13–15].

Jaw tracking technique has the potential for a lower leakage and transmission through the multi-leaf collimator (MLC) leaves by keeping jaws during dose delivery as close as possible to the MLC aperture. In this study, we evaluated the dosimetric superiority in large field IMRT or small field IMRT plans using jaw tracking technique for whole esophagus and T-shaped field radiotherapy.

## Methods and materials

### Patients and delineation

The study was approved by the ethics committee of the First Affiliated Hospital of Xiamen University. All patients provided written consent for storage of their medical information in the hospital database and for research use of this information, and the information of patients was anonymized and de-identified prior to analysis.

**Fig 1:**
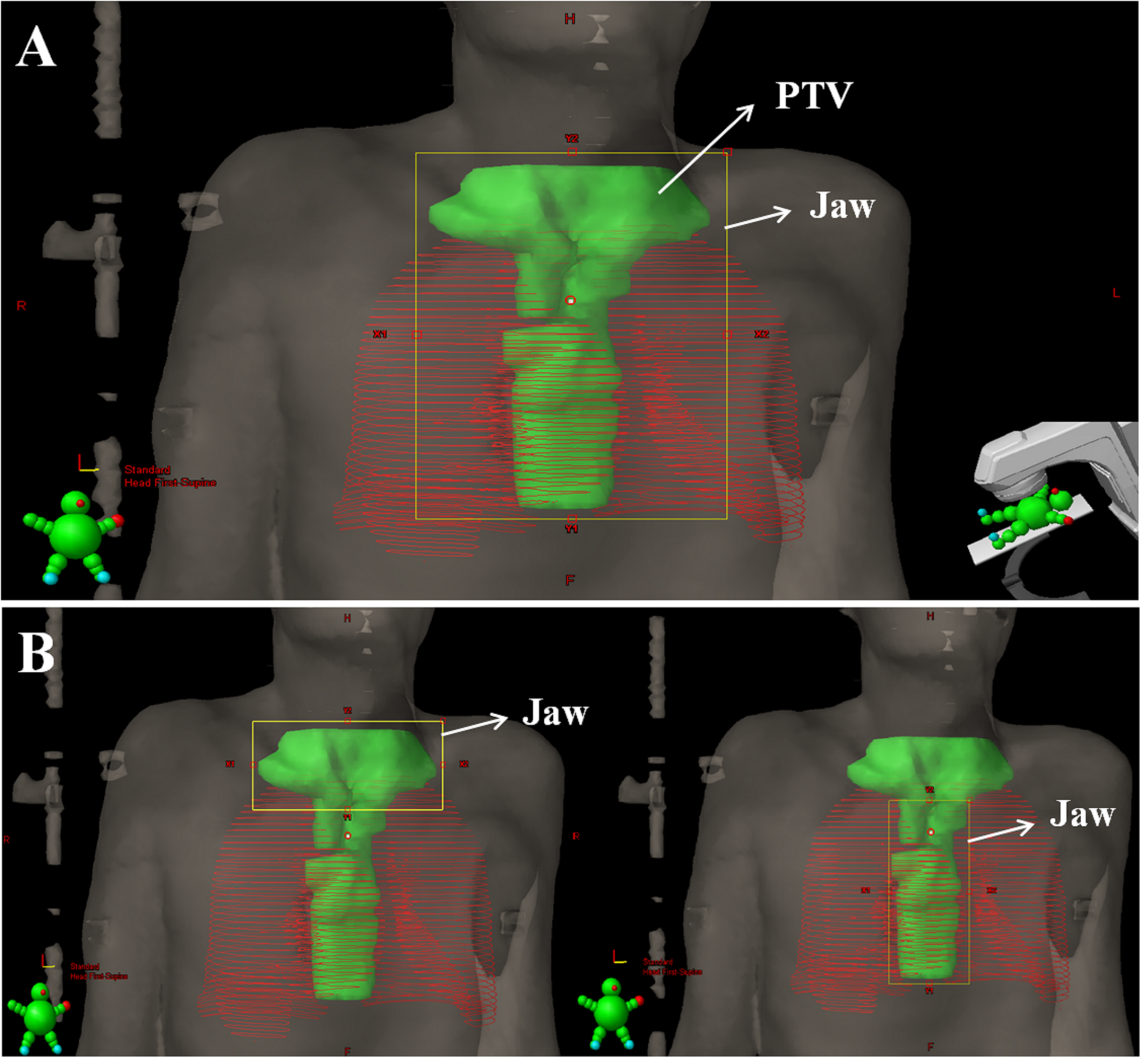
Delineated planning target volume (PTV) for esophageal cancer in beam’s eye view (BEV) and jaw setup of IMRT plans. (A) Jaw setup of large field IMRT plans. (B) Jaw setup of small field IMRT plans.

Thirty-two patients with esophageal cancer which had involvement of cervical lymph nodes were involved in this treatment planning study. The mean age was 63.9 (range, 45–84). All participants received standard of care treatment at the Department of Radiation Oncology, the First Affiliated Hospital of Xiamen University from January 2014 to October 2016. The planning CT images were acquired in 5 mm slice intervals by a planning computed tomography (General Electric Medical Systems, CT Lightspeed 16). The gross tumor volume (GTV), lymph nodes and clinical target volume (CTV) were delineated by a radiation oncologist. The CTV1 was derived from the primary tumor plus a 1 cm longitudinal expansion and 0.5 cm radial expansion. The nodal CTV1 was defined by a 0.5 cm expansion. The CTV2 was derived from the CTV1 plus a radial margin of 0.5 cm and 2 cm longitudinally. The planning target volume (PTV) was generated from CTV plus a symmetrical 5 mm margin. Fig 1 showed the T-shaped PTV in beam’s eye view (BEV) and mean PTV volume and standard deviation were 654.8 ± 161.4 cm3 (range: 389.3–971.5 cm3). Organs at risk (OAR) included the whole lungs, heart and spinal cord.

### Treatment planning techniques

Four treatment planning techniques (6F-IMRT, 6F-IMRT-T, 12F-IMRT, and 12F-IMRT-T) were generated for a TrueBeamTM linear accelerator (Varian Medical Systems, Palo Alto, CA) using 6 MV photon beam in Eclipse^®^ treatment planning system (Varian Medical Systems, AAA 11.0). For all IMRT plans, six gantry angles with coplanar beams were used and the prescribed dose (PD) to PTV was 30×2 Gy (60 Gy). Actually, cone-down technique was used in our department. Patients with esophageal cancer were irradiated with 25×2 Gy (50 Gy) to CTV2 and 30×2 Gy (60 Gy) to CTV1. For convenience purpose and more reasonable dosimetric comparison of different techniques, 60 Gy in 2 Gy fractions to PTV was used in this planning study. All IMRT plans were normalized so that 95% of PTV received 100% of the PD and to minimize the volume receiving > 107% of the PD. For every patient, gantry angles, planning objectives for PTV and normal tissues in different IMRT plans were kept constant to avoid bias.

All IMRT plans were realized for sliding window dynamic delivery with six coplanar beams. The Smart LMC Version 11.0.31 (Varian Medical Systems, Palo Alto, CA) was executed for leaf motion calculation. For the 6F-IMRT and 6F-IMRT-T plans, six large fields with six gantry angles were used (Fig 1A). Fig 1B showed the jaw setup of small field IMRT plans. Each large field was divided into two small sections in accordance with the shape of PTV (T-shaped) in BEV, thus twelve small fields were generated in 12F-IMRT and 12F-IMRT-T plans. For final dose calculation, the fixed jaw technique was selected in 6F-IMRT and 12-IMRT plans, while the jaw tracking function was applied in 6F-IMRT-T and 12F-IMRT-T plans.

### Plan evaluation and statistical methods

The quantitative evaluation of all plans was performed according to the average of the standard dose volume histograms (DVH). The values of mean dose (D_mean_), D_2%_ (dose received by 2% of the volume), D_98%_ (dose received by 98% of the volume) and V_107%_ (volume of target receiving at least 107% of the PD) were investigated for target coverage. D_98%_ and D_2%_ for PTV were defined as metrics for minimum and maximum doses. The homogeneity and conformity of plans were evaluated with homogeneity index (HI) and conformity index (CI). HI was defined as: HI defined as: HI = D_5%_/D_95%_ (dose received by 5%, and 95% of the PTV volume). CI was defined as: CI = (V_PTV_/TV_PV_)/(TV_PV_/V_TV_) [16]. V_PTV_ is the volume of PTV. TV_PV_ is the portion of PTV volume within the 100% of prescribed isodose line. V_TV_ is the volume of body that received 100% of the PD [17].

For normal tissues, the mean doses, and a set of appropriate V_x(Gy)_ and D_y(%)_ values to whole lungs, heart and spinal cord were analyzed [18–19]. The total MUs, beam on time (BOT) and mean dose rate (MU/min) were recorded to compare the delivery parameters of each technique. Statistical analyses were performed to assess the different irradiation techniques using a paired, two-tailed Wilcoxon signed-rank test. *P* ≤ 0.05 was considered statistically significant.

## Results

### PTV coverage and dose distribution

**Fig 2:**
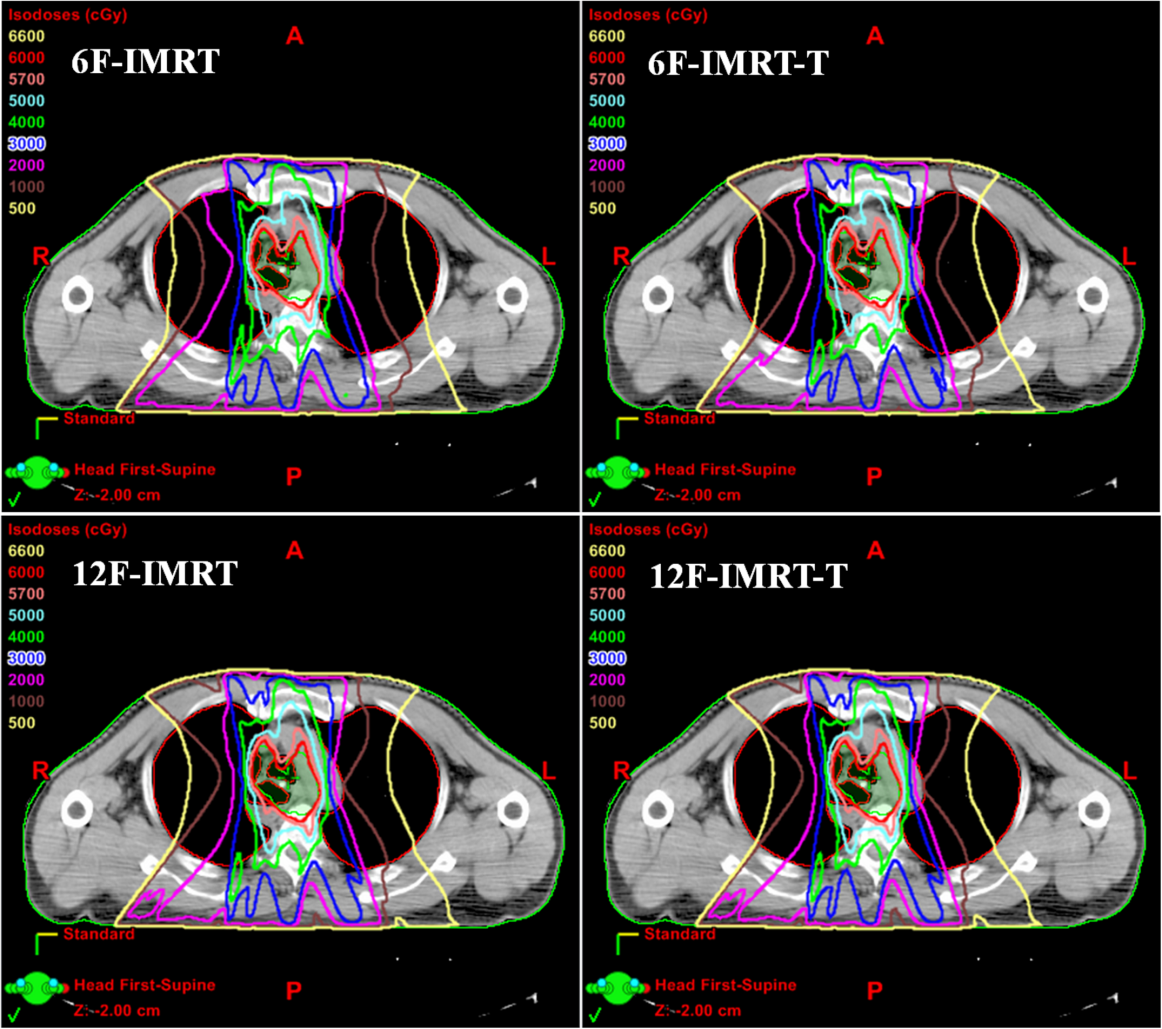
Isodose distributions for one patient with esophageal cancer in four treatment plans. 6F-IMRT = six large fields IMRT plans with fixed jaw; 6F-IMRT-T = six large fields IMRT plans with jaw tracking technique; 12F-IMRT = twelve small fields IMRT plans with fixed jaw; 12F-IMRT-T = twelve small fields IMRT plans with jaw tracking technique.

The data in Table 1 presented the dosimetric parameters of PTV for all four groups of treatment plans created with and without jaw tracking technique. Neither large field IMRT plans (6F-IMRT and 6F-IMRT-T) nor small field IMRT plans (12F-IMRT and 12F-IMRT-T) have differences in PTV coverage, homogeneity and conformity between fixed jaw and jaw tracking plans. Fig 2 showed the isodose distributions in axial views for one patient with esophageal cancer in four treatment plans. It was evident that dose distributions in 6F-IMRT-T plans were much better than 6F-IMRT plans in groups of large field IMRT plans. In groups of small field IMRT plans, 12F-IMRT plans were similar to 12F-IMRT-T plans, and both of them would further reduce normal tissue irradiation.

**Table 1:** Dosimetric parameters comparison for PTV in four treatment plans.

PTV Volume (cm^3^) = 654.8 ± 161.4, range (cm^3^) = (389.3–971.5)					
	6F-IMRT	6F-IMRT-T	12F-IMRT	12F-IMRT-T	*P* value*
D_95%_ (Gy)	60 ± 0	60 ± 0	60 ± 0	60± 0	
D_mean_ (Gy)	62.2 ± 0.4	62.4 ± 0.5	62.3± 0.5	62.3± 0.6	a<0.001, b = 0.035, c = 0.032
D_2%_ (Gy)	64.3 ± 0.6	64.5 ±0.6	64.3 ± 0.7	64.3 ± 0.8	a<0.001, b = 0.005, c = 0.955
D_98%_ (Gy)	58.8 ± 0.3	58.7 ± 0.3	58.8± 0.3	58.7 ± 0.3	a = 0.001, b = 0.001, c<0.001
V_107%_ (%)	4.8 ± 7.1	7.1 ± 9.7	5.3± 7.3	6.4 ± 9.8	a<0.001, b = 0.031, c = 0.374
CI	1.23± 0.17	1.25 ± 0.09	1.25 ±0.08	1.24 ± 0.09	a = 0.533, b = 0.958, c = 0.518
HI	1.11 ± 0.006	1.11 ± 0.01	1.09 ± 0.01	1.09 ± 0.02	a = 0.005, b = 0.032, c = 0.472

*Abbreviations*: 6F-IMRT = six large fields IMRT plans with fixed jaw; 6F-IMRT-T = six large fields IMRT plans with jaw tracking technique; 12F-IMRT = twelve small fields IMRT plans with fixed jaw; 12F-IMRT-T = twelve small fields IMRT plans with jaw tracking technique; CI = conformity index; HI = homogeneity index.

* *P* value corresponds to the paired test: a = 6F-IMRT vs 6F-IMRT-T, b = 6F-IMRT-T vs 12F-IMRT, c = 12F-IMRT vs 12F-IMRT-T.

### Dose to normal tissues

**Fig 3:**
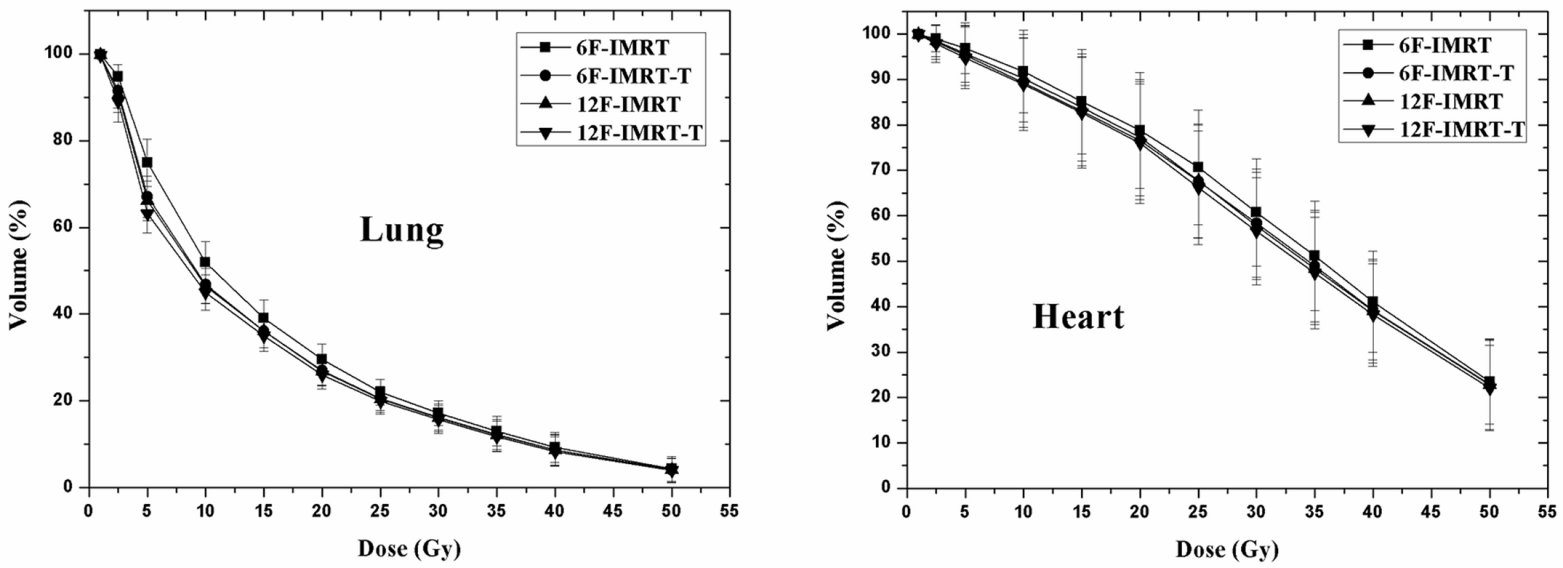
Average dose-volume histogram (DVH) comparison for lung and heart with four different planning techniques. 6F-IMRT = six large fields IMRT plans with fixed jaw; 6F-IMRT-T = six large fields IMRT plans with jaw tracking technique; 12F-IMRT = twelve small fields IMRT plans with fixed jaw; 12F-IMRT-T = twelve small fields IMRT plans with jaw tracking technique.

For radiotherapy in esophageal cancer, lung and heart are the most important organs at risk that must be considered. Fig 3 showed the average dose-volume histogram (DVH) comparison for lung and heart using four different planning techniques. Table 2 displayed the results of DVH numerical analysis for normal tissues: total lung, heart, spinal cord, and spinal cord PRV. The spinal cord PRV was derived from spinal cord plus a symmetrical 5 mm margin.

**Table 2:** Dosimetric parameters comparison for the organs at risk: Total lung, heart, spinal cord, and spinal cord PRV.

	6F-IMRT	6F-IMRT-T	12F-IMRT	12F-IMRT-T	*P* value*
Total lung Volume (cm^3^) = 3309.2 ± 735.1, range (cm^3^) = (2023–5606)					
V_2.5 Gy_ (%)	94.8± 2.7	91.5 ± 4.0	90.6 ± 4.1	88.9 ± 4.7	a<0.001, b<0.001, c<0.001
V_5 Gy_ (%)	74.9 ± 5.4	67.0 ± 4.8	66.1 ± 4.5	63.2 ± 4.5	a<0.001, b = 0.035, c<0.001
V_20 Gy_ (%)	29.5 ± 3.7	27.0 ± 3.3	26.9 ± 3.2	26.0 ± 3.2	a<0.001, b = 0.25, c<0.001
D_mean_ (Gy)	16.1± 1.6	15.0 ± 1.5	14.9 ± 1.6	14.5 ±1.6	a<0.001, b = 0.012, c<0.001
Heart Volume (cm^3^) = 646.9 ± 121.8, range (cm^3^) = (415.1–1022)					
V_5 Gy_ (%)	96.8 ± 5.6	95.2 ± 6.5	95.7 ± 6.3	94.7 ± 6.8	a<0.001, b = 0.004, c<0.001
V_40 Gy_ (%)	41.1 ± 11.1	39.2 ± 10.9	39.0 ± 11.4	38.1 ± 11.2	a<0.001, b = 0.37, c<0.001
D_mean_ (Gy)	35.2 ± 4.8	34.1 ± 4.9	34.2 ± 5.0	33.7 ± 5.1	a<0.001, b = 0.89, c<0.001
Spinal cord Volume (cm^3^) = 46.8 ± 18.3, range (cm^3^) = (22.8–95.8)					
D_max_ (Gy)	45.2 ± 1.5	44.4 ± 1.4	45.2 ±2.0	44.0 ± 1.7	a<0.001, b = 0.001, c<0.001
D_1%_ (Gy)	43.2 ± 1.4	42.4 ± 1.5	42.9 ± 1.7	41.9 ± 1.6	a<0.001, b<0.001, c<0.001
D_mean_ (Gy)	27.1 ± 5.3	26.4 ± 5.3	26.9 ± 5.3	26.2 ± 5.2	a<0.001, b<0.001, c<0.001
Spinal cord PRV Volume (cm^3^) = 158.9 ± 50.2, range (cm^3^) = (92.1–279.1)					
D_max_ (Gy)	53.4 ± 2.7	53.2 ± 2.5	53.3 ± 2.7	52.7 ± 2.7	a = 0.016, b = 0.48, c<0.001
D_1%_ (Gy)	48.4 ± 1.7	48.1± 1.6	48.3 ± 1.8	47.6 ± 1.7	a<0.001, b = 0.035, c<0.001
D_mean_ (Gy)	28.5 ± 5.0	28.6 ± 6.3	28.3 ± 4.9	27.7 ± 4.9	a<0.001, b<0.001, c<0.001

*Abbreviations*: 6F-IMRT = six large fields IMRT plans with fixed jaw; 6F-IMRT-T = six large fields IMRT plans with jaw tracking technique; 12F-IMRT = twelve small fields IMRT plans with fixed jaw; 12F-IMRT-T = twelve small fields IMRT plans with jaw tracking technique; The spinal cord PRV was derived from spinal cord plus a symmetrical 5 mm margin.

* *P* value corresponds to the paired test: a = 6F-IMRT vs 6F-IMRT-T, b = 6F-IMRT-T vs 12F-IMRT, c = 12F-IMRT vs 12F-IMRT-T.

For total lung irradiation, 6F-IMRT-T plans showed significant reduction in V5 (by 7.9%) and V20 (by 2.5%) compared to 6F-IMRT plans, and the values of V5 and V20 in 12F-IMRT-T plans could be reduced by 2.9% and 0.9% respectively compared to 12F-IMRT plans. By use of jaw tracking technique, the mean dose reduction was by 1.1 Gy (~ 6.8%) in groups of large field IMRT plans (6F-IMRT-T vs 6F-IMRT) and 0.4 Gy (~ 2.7%) in groups of small field IMRT plans (12F-IMRT-T vs 12-IMRT). With regard to heart irradiation, 6F-IMRT-T plans resulted in slight decrease in both mean dose and V40 with a reduction of 1.1 Gy (~ 3%) and 1.9% respectively compared to 6F-IMRT plans, while it was about 0.5 Gy (~ 1.5%) and 0.9% respectively for 12F-IMRT-T plans compared to 12F-IMRT plans. In the case of spinal cord, IMRT plans with jaw tracking technique also slightly reduced the values of D_1%_ compared to fixed jaw plans.

### MU and beam delivery time

Table 3 showed the number of monitor units (MU), beam-on time (BOT) and mean dose rate (MDR) for all treatment plans created with different planning techniques. In total MUs, jaw tracking plans in groups of large field IMRT resulted in about 7% increase compared to fixed jaw plans (*P* <0.001), while it was about 6% increase for groups of small field IMRT plans (*P* <0.001). Without jaw tracking technique, the small field IMRT plans were increased by average of 70.5% compared to large field IMRT plans. Regarding mean BOT, whether in groups of large field IMRT plans or in groups of small field IMRT plans, jaw tracking plans resulted in an average of 7% increase compared to fixed jaw plans (*P* <0.001).

**Table 3:** The number of monitor units (MU), beam-on time (BOT), and mean dose rate (MDR) for treatment plans created with different planning techniques.

	6F-IMRT	6F-IMRT-T	12F-IMRT	12F-IMRT-T	*P* value*
MU	1359 ± 265	1455 ± 299	2317 ± 386	2455 ± 423	a<0.001, b<0.001, c<0.001
BOT (min)	2.27 ± 0.4	2.43± 0.5	3.83 ± 0.7	4.09 ± 0.7	a<0.001, b<0.001, c<0.001
MDR (MU/min)	599.7 ± 1.6	599.9± 1.5	600 ± 1.2	600 ± 1.2	a = 0.357, b = 0.837, c = 0.695

*Abbreviations*: 6F-IMRT = six large fields IMRT plans with fixed jaw; 6F-IMRT-T = six large fields IMRT plans with jaw tracking technique; 12F-IMRT = twelve small fields IMRT plans with fixed jaw; 12F-IMRT-T = twelve small fields IMRT plans with jaw tracking technique.

* *P* value corresponds to the paired test: a = 6F-IMRT vs 6F-IMRT-T, b = 6F-IMRT-T vs 12F-IMRT, c = 12F-IMRT vs 12F-IMRT-T.

## Discussion

The dosimetric study addressed a comparative appraisal of the role of IMRT using jaw tracking technique in whole esophagus and T-shaped field radiotherapy for advanced esophageal cancer. For better normal tissue sparing, CTV2 was prescribed to 60 Gy in the treatment plan. This would over-estimate the lung dose compared with a real-world treatment plan. However, it did not affect the dosimetric comparison of different techniques in our study.

In this study, plan quality and efficiency were assessed in both large field IMRT plans and small field IMRT plans with or without jaw tracking technique. The data showed in this paper demonstrated that jaw tracking plans resulted in better plans with respect to normal tissues sparing compared to fixed jaw plans.

A multileaf collimator (MLC) for photon beams in accelerator consists of a large number of leaves that can be driven automatically to generate a field of any shape for radiotherapy techniques. Varian TrueBeam linac was equipped with standard Millennium MLC with 120 leaves (0.5 cm spatial resolution at isocenter in the inner 20 cm and 1.0 cm spatial resolution for the 2×10 cm outer length of the field). The interleaf (between sides) transmission cannot be ignored in IMRT, although the transmitted dose rate is usually less than 3%. Due to the MLC transmission, more irradiation in normal tissues such as lung and heart may be occurred in IMRT for esophageal cancer. However, the primary beam transmission would be further minimized by combining jaws with the MLC in shielding areas outside the MLC field opening, and the transmitted dose rate could be 0.90%–4.40% (6 MV photon) or 1.14%–7.00% (18 MV) lower than that shielded only by MLC [20]. The study aimed to compare the jaw tracking technique with the fixed jaw technique on the dosimetry. The fixed jaw plans was first to generate in Eclipse treatment planning system and subsequently selected jaw tracking options at final dose calculation to create the jaw tracking plans. For esophageal cancer radiotherapy using IMRT technique, the jaw tracking plans resulted in lower volumes in low-dose regions (about 0–20 Gy) especially in terms of lung V5 compared to fixed jaw plans (Table 2). The value of V5 in total lung is causing more and more attentions by radiation oncologist for its role in the occurrence of radiation pneumonitis [21–22]. Therefore, the jaw tracking technique may have potential for reducing radiation-induced toxicity from transmitted radiation through MLC.

During clinical radiotherapy of thoracic cancer, the planning objectives for lung were usually a volume receiving ≥ 5 Gy not more than 65%, volume receiving 20 Gy < 30% and the mean dose < 15 Gy [11]. It was a challenge to generate IMRT plans in accordance with the dosimetric objectives for whole esophagus and T-shaped field radiotherapy due to the large targets (in this study target volume was 654.8 cm^3^ on average, ranging from 389.3 cm^3^ to 971.5 cm^3^). With respect to the values of V5 and mean dose for total lung, we observed that even jaw tracking plans cannot meet the objectives in groups of large field IMRT plans. To further reduce lung irradiation in low-dose regions, we adopt small field IMRT technique to conform the T-shaped PTV as shown in Fig 1B. The small field IMRT plans resulted in lower values of lung V5 (by 8.8%) compared to large field IMRT plans, even less than large field IMRT plans with jaw tracking plans (by 0.9%). This can be attributed to the decrease of MLC transmitted dose in small field IMRT plans. In addition, by the use of a combination of small field IMRT and jaw tracking techniques, the 12F-IMRT-T plans showed best plans among these four treatment plans with the possibility to further reduce the dose delivered to lung. In this case, the mean value of V5 for total lung in 12F-IMRT-T plans was only 63.2%, which can meet the planning objectives for lung.

## Conclusion

In summary, we explored various possible physics improvements for whole esophagus and T-shaped field radiotherapy with IMRT technique. The results demonstrate that small field IMRT plans showed superiority in lung and heart sparing compared to large field IMRT plans, while the small field IMRT plan with jaw tracking technique resulted in further normal tissues sparing compared to fixed jaw plans.
